# Global burden, trends, and inequalities of ischemic heart disease among young adults from 1990 to 2019: a population-based study

**DOI:** 10.3389/fcvm.2023.1274663

**Published:** 2023-11-24

**Authors:** Peng Wu, Shuixiu Yu, Jun Wang, Shenglan Zou, De-Shan Yao, Yuan Xiaochen

**Affiliations:** ^1^ Department of Cardiology, Affiliated Hospital of Yangzhou University, Yangzhou, China; ^2^ Department of Oncology, Jingjiang People’s Hospital, Taizhou, China

**Keywords:** ischemic heart disease, GBD 2019, young adults, health inequality, disability-adjusted life-years (DALYs)

## Abstract

**Background:**

Ischemic heart disease (IHD) is a major global health concern, and its burden among young adults aged 25–49 years remains underexplored. This study aims to provide a comprehensive assessment of the global burden and trends of IHD over the past 30 years (1990–2019) among this age group, as well as to analyze the health inequalities related to socioeconomic development.

**Methods:**

Data from Global Burden of Disease Study 2019 (GBD 2019) were utilized to analyze the prevalence, mortality, and disability-adjusted life years (DALYs) rate of IHD among young adults globally. Joinpoint regression analysis was applied to examine the trends over the study period. Health inequality analysis was performed to investigate the disparities in IHD burden related to the Socio-Demographic Index (SDI) of countries.

**Results:**

According to GBD 2019 data, in 2019, the global numbers of young adults with IHD cases, deaths, and DALYs were 18,050,671 (95% UI, 15,551,940–21,254,746), 597,137 (548,250–647,778), and 28,692,968 (26,397,448–31,178,464), respectively, accounting for 9.15%, 6.53%, and 15.7% of the total global cases. Over the past 30 years, the mortality [AAPC = −0.4%, 95% CI (−0.7% to −0.1%)] and DALYs rate [AAPC = −0.3%, 95% CI (−0.6% to −0.1%)] of IHD among young adults decreased, while the prevalence rate [AAPC = 0.4%, 95% CI (0.4%–0.4%)] and YLDs rate [AAPC = 0.4%, 95% CI (0.3%–0.4%)] increased. Furthermore, countries with lower levels of socio-demographic index (SDI) disproportionately bore a higher burden of IHD among young adults. The inequality slope index for young adult IHD shifted from −56.6 [95% CI (−480.4–370.2)] in 1990 to −583.0 [95% CI (−996.8 to −169.2)] in 2019, and the concentration index moved from −8.2 [95% CI (−8.5 to −7.9)] in 1990 to −13.2 [95% CI (−13.9 to −12.4)] in 2019.

**Conclusions:**

While the mortality and DALYs rate of IHD among global young adults have decreased over the past 30 years, the degree of inequality related to SDI among countries has continued to increase. Decision-makers in various countries should allocate resources wisely and implement effective strategies to improve the burden of young adults IHD globally and address the health inequalities associated with it.

## 1. Introduction

Ischemic heart disease (IHD) refers to heart diseases caused by coronary artery stenosis or occlusion due to atherosclerosis, leading to myocardial ischemia or necrosis, which seriously endangers human health. Since 1990, the total number of disability-adjusted life years (DALYs) and deaths from IHD has steadily increased, making it the leading cause of global deaths by 2019, with over 180 million DALYs and 9 million deaths (1). IHD remains one of the major public health threats, and recent studies have estimated the continuously increasing global burden of IHD (2).

IHD has become the “number one killer” for individuals over 50 years old (3), but its impact on younger people (below 50 years old) cannot be ignored. Common traditional risk factors for IHD, including obesity and diabetes, have shown a significant increase in global prevalence (4, 5). Additionally, lack of awareness about IHD and its related risk factors among young people, along with new issues such as social relationships, psychological distress, and insufficient sleep (less than 6 h per night) have also contributed to the occurrence of IHD among the younger population (6, 7). Currently, there is a lack of comprehensive research and reporting on the global burden of IHD in young populations. Given that premature mortality among the young and middle-aged will have a significant impact on society, this study aims to use the Global Burden of Disease (GBD) database (1990–2019) to analyze the global burden, trends, and health inequalities of IHD, and help countries develop public health policies, prioritize the management of IHD in the young adults, and reduce the increasing financial and health burden caused by IHD among young adults.

## 2. Methods

### 2.1. Data source

The GBD 2019 utilized the latest epidemiological data and improved standardized methods coordinated by the Institute for Health Metrics and Evaluation (IHME). As a continuous quality improvement process, IHME updates each GBD study annually by incorporating all known advances in data, modeling, estimation methods, and health knowledge to reestimate the entire time series, ensuring that each GBD study includes the latest estimates. This study is based on the data obtained from the GBD 2019, which includes the annual comprehensive assessment of 369 diseases, injuries, and disabilities and 87 risk factors from 204 countries and territories worldwide from 1990 to 2019 (3, 8). In this study, young adults are defined as individuals aged 25–49 years, who are typically at the peak of physical health and are the primary workforce in society. We extracted the estimated numbers of deaths, prevalence, DALYs, and years lived with disability (YLDs) of IHD among the 25–49 age group globally and in 204 countries and territories from GBD 2019, along with their 95% uncertainty intervals (UIs), as the indicators for measuring IHD. We also used the Socio-demographic Index (SDI), a comprehensive index based on per capita income, total fertility rate for women under 25 years old, and average years of education for adults aged 15 and above, to represent the socioeconomic level. The SDI ranges from 0 to 1, with higher values indicating higher socioeconomic levels.

### 2.2. Statistical analysis

In this study, we employed joinpoint regression models to analyze the temporal trend changes in the burden of IHD among young adults. Joinpoint regression is a group of statistical linear models that use segmented linear regression to identify trends represented by one or more segments (9). We reported the annual percent change (APC) and its 95% confidence interval (CI) for the entire period from 1990 to 2019. The joinpoint regression model identified statistically significant joinpoints representing trend changes. Bayesian Information Criterion was used to identify the best-fitting model for the data (10). If the APC differed significantly from 0, it indicated an increasing (worsening) or decreasing (improving) trend. If it did not differ from zero, the trend was defined as stable or flat. We also evaluated the average annual percent change (AAPC) and its corresponding 95% CI, which is a summary measure of the trend over a pre-specified fixed interval, calculated as the weighted average of the annual percent changes, with the weights being the lengths of the intervals. Joinpoint trend analysis was performed using the “Joinpoint” software (version 4.9.1.0) from the Surveillance Research Program of the U.S. National Cancer Institute. The significance level for *p*-values was set at 0.05.

### 2.3. Cross-country analysis of health inequality

In this study, we used the slope index of inequality and concentration index to quantify the distributional inequalities of IHD burden among countries, which represent absolute and relative gradient inequalities, respectively (11). The slope index of inequality was calculated by regressing the country DALYs rates on a relative index scale related to SDI, defined by the midpoint of the range of the population accumulated by SDI ranking. Heteroscedasticity was addressed using weighted regression models. The concentration index was calculated by numerically integrating the area under the Lorenz concentration curve, which used the cumulative fraction of DALYs and the cumulative relative distribution of the population ranked by SDI (12). All analyses and visualizations were performed using the World Health Organization's Health Equity Assessment Toolkit (HEAT and HEAT Plus) and R software (V.4.2.3).

### 2.4. Ethical statement

The GBD research used established data and was reviewed and approved by the Institutional Review Board of the University of Washington with a waiver of informed consent. All information regarding ethical standards can be obtained from the official website (http://www.healthdata.org/gbd/2019). This study did not involve individual subjects.

## 3. Results

### 3.1. Global burden of IHD among young adults in 2019

**Figure 1:**
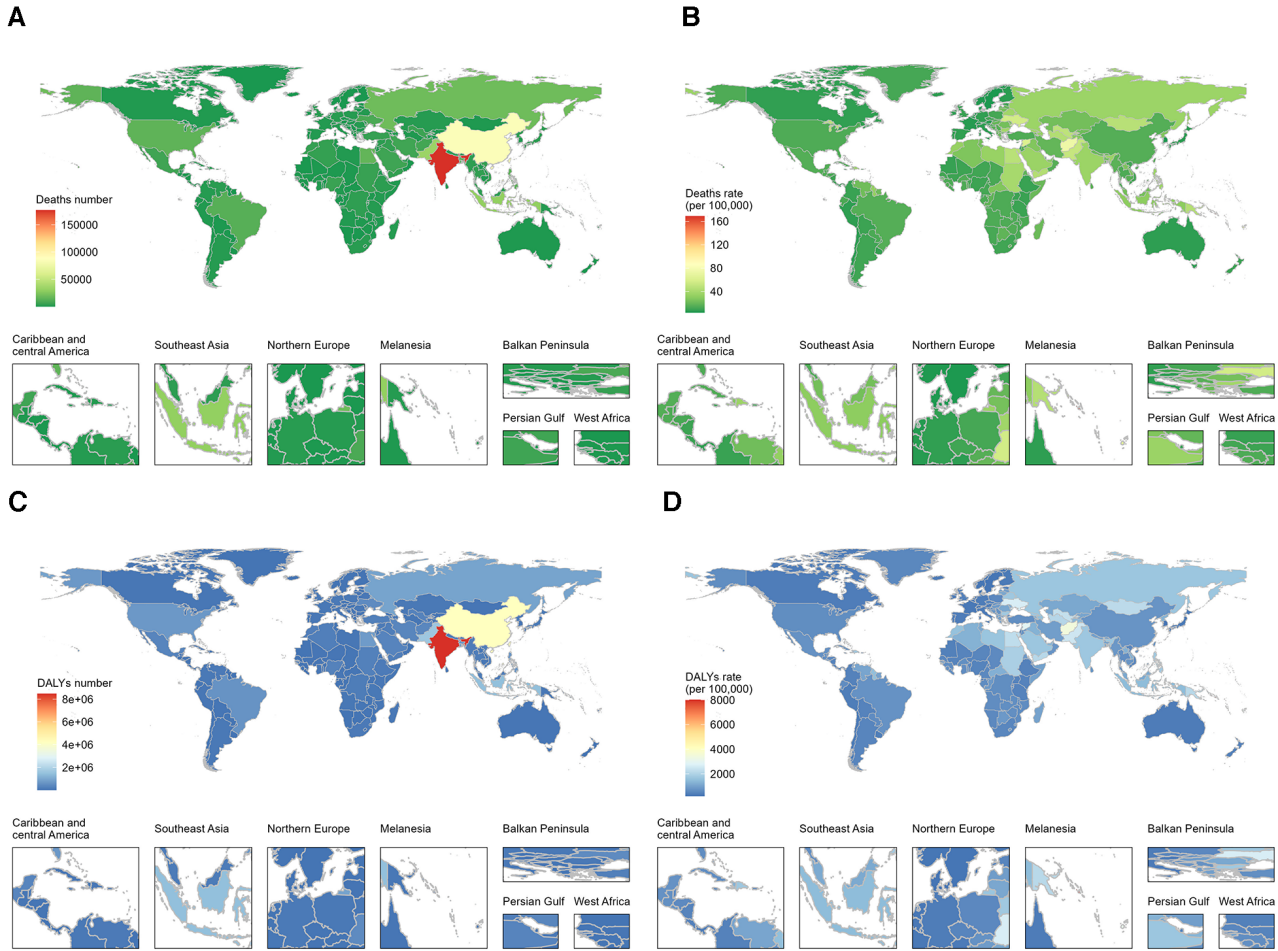
Global burden of ischemic heart disease (IHD) among young adults in 204 countries and territories in 2019, depicted through maps for deaths number (**A**), deaths rate (**B**), DALYs number (**C**), and DALYs rate (**D**).

In 2019, there were a total of 197 million cases of IHD globally, with 9.14 million deaths, and young adults aged 25–49 accounted for 9.15% of the prevalence cases and 6.53% of the IHD-related cardiovascular deaths (Table 1). Ischemic heart disease in the 25–49 age group led to 597,137 (95% UI, 548,250–647,778) deaths and 28,692,968 (95% UI, 26,397,448–31,178,464) DALYs. The top three countries with the heaviest absolute burden were India (deaths: 176,711; DALYs: 8,477,941), China (deaths: 85,231; DALYs: 4,218,185), and Pakistan (deaths: 33,942; DALYs: 1,642,499) (Figures 1A,C). The corresponding death rates and DALYs rates were 35.6 and 1,710.1 per 100,000 population, 15.1 and 748.3 per 100,000 population, and 51.2 and 2,491.4 per 100,000 population, respectively. The countries with the highest death rates mainly concentrated in some low-income South Pacific island nations, with the Solomon Islands (death rate: 169.0/100,000; DALYs rate: 8,014.8/100,000), Nauru (death rate: 123.5/100,000; DALYs rate: 5,910.8/100,000), and Kiribati (death rate: 99.0/100,000; DALYs rate: 4,762.9/100,000) ranking in the top three (Figures 1B,D). In contrast, Israel, South Korea, Denmark, and Switzerland showed significantly lower disease burden in young adults for IHD (death rate <4.4/100,000; DALYs rate <204.0/100,000).

**Table 1:** Prevalence, mortality, and DALYs of ischemic heart disease (IHD) worldwide in 2019, along with their trends from 1990 to 2019.

	All ages	Aged 25–49 years
2019		
Mortality		
Number	9,137,791 (8,395,682–9,743,550)	597,137 (548,250–647,778)
Rate (per 100,000)	118.1 (108.5–125.9)	22.0 (20.2–23.9)
Prevalence		
Number	197,219,450 (17,768,8201–219,501,075)	18,050,671 (15,551,940–21,254,746)
Rate (per 100,000)	2,548.9 (2,296.5–2,836.9)	664.7 (572.7–782.7)
DALYs		
Number	182,030,144 (170,206,778–193,504,630)	28,692,968 (26,397,448–31,178,464)
Rate (per 100,000)	2,352.6 (2,199.8–2,500.9)	1,056.7 (972.1–1,148.2)
AAPC from 1990 to 2019		
Prevalence	1.2% (1.2–1.2)	0.4% (0.4–0.4)
Mortality	0.4% (0.2–0.5)	−0.4% (−0.7 to −0.1)
DALYs	0.1% (0–0.3)	−0.3% (−0.6 to −0.1)
YLDs	1.2% (1.1–1.2)	0.4% (0.3–0.4)

### 3.2. Trends in IHD prevalence, mortality, and burden from 1990 to 2019

**Figure 2:**
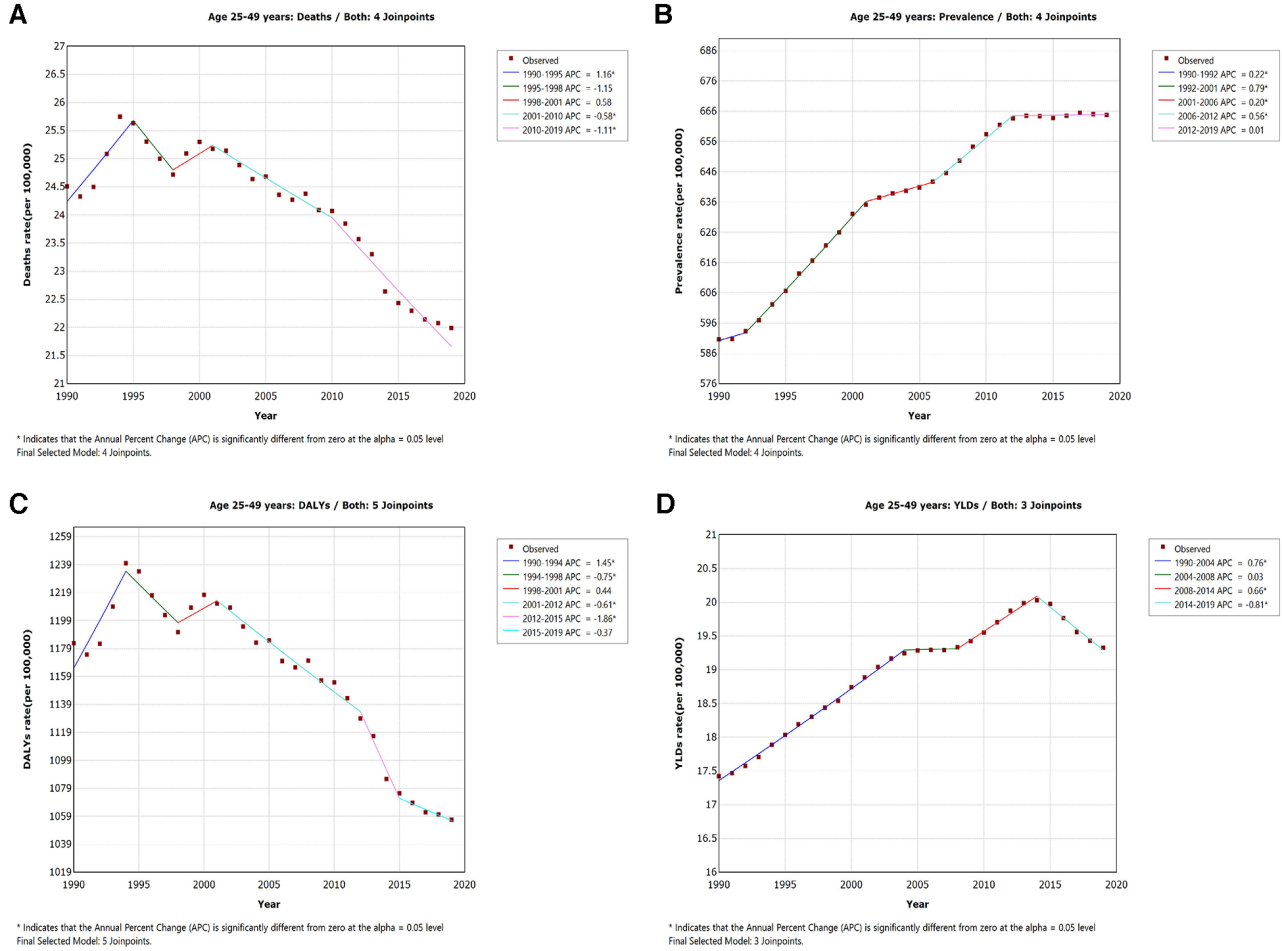
APC in IHD mortality (**A**), prevalence (**B**), disability-adjusted life-years (**C**), and years lived with disability (**D**) per 100, 000 population among young adults globally from 1990 through 2019.

Over the past 30 years, there have been overall increases in the prevalence, mortality, DALYs, and YLDs rate of IHD among the global population of all age groups, with AAPC values of 1.2%, 0.4%, 0.1%, and 1.2%, respectively (*p* < 0.05) (Table 1). However, for the 25–49 age group, from 1990 to 2019, the rates of prevalence and YLDs of IHD increased overall, while the mortality and DALYs rate decreased. More specifically, as shown in Table 1 and Figure 2, the prevalence rate of IHD per 100,000 population among the 25–49 age group increased from 590.6 to 664.7 annually (AAPC =  + 0.4%, *p* < 0.05). The most significant increase occurred between 1992 and 2001 [APC = 0.8%, 95% CI (0.8%–0.8%), *p* < 0.05], and there was stability in the most recent seven years (from 2012 to 2019). In contrast, over the past 30 years, the mortality of IHD per 100,000 population among the 25–49 age group decreased from 24.5 to 22.0 (AAPC = −0.4%, *p* < 0.05). The most significant decrease occurred between 2010 and 2019 [APC = −1.1%, 95% CI (−1.4% to −0.8%), *p* < 0.05], while there was an increase from 1990 to 1995 [APC = 1.2%, 95% CI (0.6%–0.7%), *p* < 0.05]. Similarly, in terms of DALYs, the DALYs rate per 100,000 population of IHD cases among the 25–49 age group decreased overall from 1,182.9 to 1,056.7 (AAPC = −0.3%, *p* < 0.05). The most significant decrease occurred between 2012 and 2015 [APC = −1.9%, 95% CI (−3.6%–−0.1%), *p* < 0.05], with stability between 2015 and 2019. However, the YLDs rate of IHD among young people showed a different trend. As shown in Figure 2D, the YLDs rate per 100,000 population of IHD among the 25–49 age group increased overall from 17.4 to 19.3 (AAPC = 0.4%, *p* < 0.05) from 1990 through 2019. The most significant increase occurred between 1990 and 2004 [APC = 0.8%, 95% CI (0.7%–0.8%), *p* < 0.05], and a decreasing trend was observed between 2014 and 2019 [APC = −0.8%, 95% CI (−0.9% to −0.7%), *p* < 0.05].

### 3.3. Analysis of health inequalities among young adults with IHD

**Figure 3:**
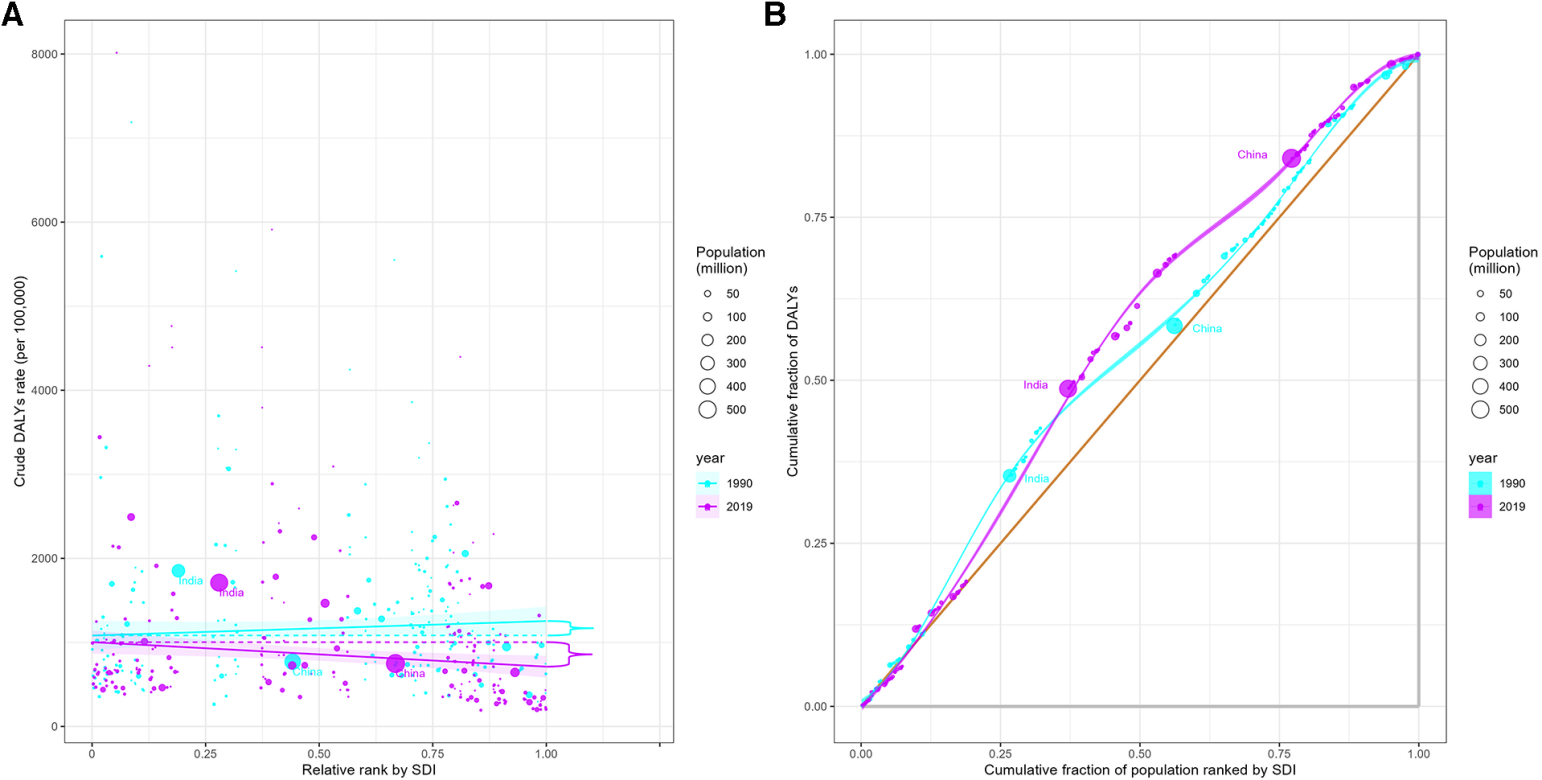
Health inequality regression curves (**A**) and concentration curves (**B**) for the DALYs in IHD among young adults, 1990 and 2019.

In the 204 countries and territories worldwide, significant absolute and relative inequalities related to SDI were observed in the burden of IHD among young adults. Countries with lower SDI levels bore a disproportionately higher burden. As shown in Figure 3, the inequality slope index for DALYs rate between countries with the highest and lowest SDI values was −56.6 [95% CI (−480.4–370.2)] in 1990, and this inequality gap significantly increased to −583.0 [95% CI (−996.8 to −169.2)] in 2019. Moreover, the concentration index, which measures relative gradient inequalities, was −8.2 [95% CI (−8.5 to −7.9)] in 1990 and −13.2 [95% CI (−13.9 to −12.4)] in 2019.

## 3. Discussion

In this study, we utilized the global data from GBD 2019 to provide a comprehensive assessment of the burden of IHD among young adults aged 25–49 globally and its trends over the past 30 years. It has been well-established that the consequences of premature IHD are devastating. It has been reported as the leading cause of death and premature death in various populations across different states in the United States (13). India and China are two populous countries and emerging economies that have reaffirmed their political commitment to prevention and control of non-communicable diseases, including IHD, in 2013, and made efforts to reduce premature mortality from cardiovascular diseases thereafter (14–16). Our analysis also showed that the burden of IHD among young adults in these two populous countries has improved to some extent, with relatively lower ranks for mortality and DALYs, although the absolute burden remains high. Low-income South Pacific island nations such as the Solomon Islands, Nauru, and Kiribati, despite having small population bases, have extremely high mortality and DALYs rates of IHD among young adults. Among the top 10 countries with the highest mortality and DALYs rates, nine are from the South Pacific islands. Due to limited land area, dispersed locations, and distance from the center of global economic and social activities, these South Pacific island countries face challenges in developing basic healthcare facilities. In 2013, the United Nations adopted measures to reduce behavioral and biological risks to achieve a 25% reduction in cardiovascular disease mortality by 2025, and in 2021, they developed a roadmap for implementing the Global Action Plan for the Prevention and Control of Noncommunicable Diseases 2023–2030 (17). Clearly, more international cooperation and assistance are needed for these low-income South Pacific island countries. Perhaps most striking was that, as a country with both very high absolute and relative burdens, Pakistan was also experiencing the population growth and infectious diseases (18, 19). Preventing and treating young adults with IHD may be another important public health challenge facing the Pakistani government and health sector.

The long-term impact of IHD on survivors can lead to economic hardships and functional impairments, causing devastating consequences for young patients and their families (20). Currently, there are few reports addressing the incidence, prevalence, and trends of young adult IHD burden. Some small-scale studies have reported stable or slightly increasing rates of myocardial infarction in young patients across multiple countries (21–23). A study conducted in two hospitals in Beijing, China also reported a significant increase in the number of patients under 45 years old with coronary artery disease from 2010 to 2014 (24). In summary, several studies suggest a stable or increasing trend in the incidence of coronary artery disease in patients under 50 years old, while the incidence in elderly individuals is declining (25). Our study provides a comprehensive analysis of the long-term trends in the rate of prevalence, mortality, DALYs, and YLDs of IHD among young adults globally from 1990 to 2019. Over the past 30 years, there were overall increases in the prevalence, mortality, DALYs, and YLDs rate of IHD among the global population of all age groups. However, for the 25–49 age group, the trends were different, with an increase in prevalence and YLDs rate but a decrease in mortality and DALYs rate. Figure 2 showed that although the rate of prevalence and YLDs among young adults increased from 1990 to 2019, the prevalence, mortality, DALYs and YLDs have decreased or remained stable in the last 5 years. These findings may be attributed to improved early diagnosis and better specialized treatment in recent years. Additionally, young adults tend to have better compliance with medical treatments and lifestyle interventions, leading to improved disease management and prevention. However, some studies predict that the number of IHD deaths in both men and women will continue to increase in the next 20 years, with an estimated 15.8 million deaths due to IHD by 2039, posing significant challenges to human health and social development (26). Governments of different countries and regions need to implement more effective measures to further reduce the global burden of IHD.

Quantifying the inequalities in the burden of IHD among young adults related to SDI can shed light on the distribution patterns of disease burden based on the level of socioeconomic development. The Prospective Urban Rural Epidemiologic (PURE) study indicates that major cardiovascular events are more common in lower-income countries and populations with lower education levels (27). Compared to high-income countries, cardiovascular diseases have higher incidence and mortality rates in middle- and low-income countries, despite the presence of more risk factors for cardiovascular diseases in high-income countries (28). This also suggests that the high mortality rate in poorer countries may not be directly related to risk factors but could be attributed to poorer access to healthcare (28). Further research also suggests the need to actively enhance the prevention and control of cardiovascular diseases in both women and men, especially in middle- and low-income countries (29). Our study arrives at similar conclusions regarding young adults IHD. Using the standard health equity analysis method recommended by the World Health Organization, we conducted an analysis of inequality between countries for young adults IHD and found that countries with lower SDI bear a disproportionately high burden of IHD. Furthermore, over time, the degree of inequality related to SDI in the burden of IHD among young adults has increased overall. This suggests that over the past 30 years, investment in the prevention, management, and treatment of IHD among young adults may have been insufficient as socioeconomic development levels have improved. The predicted increase in disease burden should prompt healthcare decision-makers to pay greater attention to IHD among young adults, especially in low-SDI countries, and to strengthen primary healthcare and primary prevention of IHD. Measures such as smoking cessation, reducing salt intake, early treatment of hypertension, and repeated interventions for healthy eating habits during childhood can further reduce premature deaths from IHD among young adults (30, 31).

To the best of our knowledge, this study represents the latest and most comprehensive analysis of the global burden of IHD among young adults aged 25–49 years over the past 30 years (1990–2019). The main strengths of this study include its long-term observation, wide geographical coverage, and large dataset. Despite significant efforts to provide a comprehensive analysis, the study has some limitations. Firstly, cases in low-income countries in the GBD may be underestimated, as there are limitations in diagnosis or reporting in low-income countries due to their poor healthcare performance, which may lead to misdiagnosis, underdiagnosis, file losses, and so on. Secondly, our study heavily relies on secondary data, and the results based on GBD modeling may be subject to inaccuracies and incompleteness in real-world data sources. Additionally, race and genetic factors may play a role in the distribution of IHD among different regions and countries (32, 33). However, data on different racial populations are not available through GBD studies, and therefore, race information needs to be collected and analyzed in future studies.

## 5. Conclusions

In summary, our study provides a comprehensive assessment of the distribution of burden and its changing patterns of IHD among young adults aged 25–49 globally over the past 30 years. Although the deaths and DALYs burden of IHD among young adults has decreased over the past 30 years globally, we found that countries with lower levels of socioeconomic development have borne a disproportionately higher burden of IHD. The increasing degree of inequality related to socioeconomic development over time further emphasizes the importance of increased investment in cardiovascular health for young adults, especially in low-SDI countries. We hope that this study will provide detailed information for policymakers to allocate resources, implement effective measures, promote cooperation among countries, and improve the burden of IHD among young adults in low-income countries.
